# Investigating Default Mode and Sensorimotor Network Connectivity in Amyotrophic Lateral Sclerosis

**DOI:** 10.1371/journal.pone.0157443

**Published:** 2016-06-20

**Authors:** Sneha Chenji, Shankar Jha, Dawon Lee, Matthew Brown, Peter Seres, Dennell Mah, Sanjay Kalra

**Affiliations:** 1 Neuroscience and Mental Health Institute, University of Alberta, Edmonton, Canada; 2 Department of Psychiatry, University of Alberta, Edmonton, Canada; 3 Department of Biomedical Engineering, University of Alberta, Edmonton, Canada; 4 Division of Neurology, University of Alberta, Edmonton, Canada; 5 Department of Medicine, University of Alberta, Edmonton, Canada; Center of Genomic & Post Genomics, ITALY

## Abstract

Amyotrophic lateral sclerosis (ALS) is a neurodegenerative condition characterized by degeneration of upper motor neurons (UMN) arising from the motor cortex in the brain and lower motor neurons (LMN) in the brainstem and spinal cord. Cerebral changes create differences in brain activity captured by functional magnetic resonance imaging (fMRI), including the spontaneous and simultaneous activity occurring between regions known as the resting state networks (RSNs). Progressive neurodegeneration as observed in ALS may lead to a disruption of RSNs which could provide insights into the disease process. Previous studies have reported conflicting findings of increased, decreased, or unaltered RSN functional connectivity in ALS and do not report the contribution of UMN changes to RSN connectivity. We aimed to bridge this gap by exploring two networks, the default mode network (DMN) and the sensorimotor network (SMN), in 21 ALS patients and 40 age-matched healthy volunteers. An UMN score dichotomized patients into UMN+ and UMN- groups. Subjects underwent resting state fMRI scan on a high field MRI operating at 4.7 tesla. The DMN and SMN changes between subject groups were compared. Correlations between connectivity and clinical measures such as the ALS Functional Rating Scale—Revised (ALSFRS-R), disease progression rate, symptom duration, UMN score and finger tapping were assessed. Significant group differences in resting state networks between patients and controls were absent, as was the dependence on degree of UMN burden. However, DMN connectivity was increased in patients with greater disability and faster progression rate, and SMN connectivity was reduced in those with greater motor impairment. These patterns of association are in line with literature supporting loss of inhibitory interneurons.

## Introduction

Amyotrophic Lateral Sclerosis (ALS) is a progressive neurodegenerative disease with death occurring within 2 to 5 years [1]. It has a lifetime risk of 1/400 with a rising incidence due to the aging population [2]. Progressive disability in ALS is due to muscular weakness caused by degeneration of the upper motor neurons (UMN) in the motor cortex and of the lower motor neurons (LMN) in the brain stem and spinal cord. Frontotemporal lobar degeneration (FTLD) is an additional pathological feature observed in ALS resulting in cognitive impairment in upwards of 50% of patients; in 10–15% of patients the impairments are severe enough to meet criteria for frontotemporal dementia (FTD) [3].

These aforementioned motor and cognitive changes in ALS may be reflected as changes in baseline neuronal activity in the brain. In the past decade, there has been a surge of studies investigating the neurodegenerative changes in ALS using magnetic resonance imaging [MRI]. Resting state functional MRI (rs-fMRI) techniques have enabled non-invasive investigation of regional brain interactions. It captures low frequency (0.01–0.1 Hz) blood oxygen level dependent (BOLD) signals that reflect spontaneous neuronal activity in cortical and sub-cortical regions at rest, when the brain is not particularly occupied with a goal-oriented task. These spontaneous activities in anatomically separated regions form resting state networks (RSNs) that reflect a level of on-going functional activity between brain regions, and serve as the foundation for higher order motor and cognitive functions [4, 5].

Studies report that RSNs are consistent across healthy participants and are highly reproducible [6–9]. In contrast to task-based fMRI, rs-fMRI is task-free and therefore is accessible to patients who cannot perform explicit tasks requiring motor functions. In addition to its larger application, resting state studies offers a new perspective on neurodegenerative disorders. It helps to understand the activity of the degenerating brain in baseline conditions, as it is believed to characterize large-scale system integrity [10]. The current paper refers to functional connectivity in terms of the strength of association between anatomically separate brain regions with one another. Our study specifically focuses on the analysis of RSN functional connectivity in patients with ALS with a primary focus on the default mode network (DMN) and sensorimotor network (SMN).

The aforementioned networks were initially reported in healthy subjects in 1995 [11]. The default mode network (DMN) is considered the baseline functional network when an individual is not engaged in a specific goal oriented task. The activity in the DMN decreases when performing a goal oriented task. Correspondingly, the DMN is negatively correlated with various RSNs that are related to specific tasks such as motor functions (SMN). Previous studies in ALS have identified either increased [12–14], decreased [15, 16] or no change [17] in DMN connectivity.

The SMN includes somatosensory (postcentral gyrus) and motor (precentral gyrus) regions and extends to the supplementary motor areas. Studies have indicated that this network is activated during motor tasks such as finger tapping [11] indicating that these regions may involve a pre-mediated state that ready the brain in performing/co-ordinating a motor task. Studies in ALS have reported either lower SMN connectivity in the premotor cortex (PMC) [15–18] or higher connectivity in the PMC that is usually associated with loss of white matter integrity in the corpus callosum and corticospinal tract [19, 20].

While lower connectivity has been associated with lower grey matter volumes [16, 17], reports of higher connectivity suggest possible compensatory mechanisms in the disease course of ALS. While some studies report associations between DMN and SMN connectivity and the ALS functional rating scale–revised (ALSFRS-R) [12, 18, 19, 21], a few studies did not find any significant association between impaired connectivity and disease severity as inferred by the ALSFRS-R [14, 16]. Studies also report that RSN connectivity changes may be associated with other clinical parameters such as disease duration [14, 18, 22], and disease progression rate [14, 18, 22, 23].

Additionally, there is limited literature on the role of UMN disease burden and functional changes in ALS. Studies report that structural measures for white matter (WM) integrity such as fractional anisotropy (FA) and mean diffusivity (MD) may predict UMN disease burden in ALS patients [24]. To our best knowledge the role of UMN disease burden has not been explored with RSN connectivity in ALS. As RSN changes in ALS are often associated with structural changes [16, 17, 20, 23], we attempted to explore if RSN is altered in patients with higher UMN disease burden.

We hypothesized that (1) there will be impaired connectivity in both the DMN and SMN in ALS patients as compared to controls; (2) patients with higher UMN disease burden will have more impairments in connectivity as compared to patients with lower UMN disease burden; and (3) impaired RSN connectivity in patients will be associated with higher disease severity, longer symptom duration, increased disease progression rates and higher UMN disease burden.

## Methods

### Sample

We recruited 21 ALS patients (15 males; 6 females) from the multidisciplinary ALS clinic at the University of Alberta. All patients met research criteria for possible, probable, or definite ALS [25] and thus had both LMN and UMN signs on neurological examination. We also recruited 40 age matched healthy controls (18 males; 20 females) who were either spouse/caregivers of the patients or volunteers who learned of the study by word of mouth. Our final analysis included only 20 patients and 34 controls (Table 1). Details on exclusion of subjects from analysis are described in the Voxel-wise Correlations section in Methods. Ethics approval was provided by the Health Research Ethics Board of the University of Alberta and written informed consent was obtained from all participants.

**Table 1:** Clinical profile of participants.

Variable	Patients (n = 20)	Controls (n = 34)	*p*
Gender (M / F)^‡^	14 / 6	15 / 19	0.06
Age (years)	57.1 ± 13.5	54.4 ± 13.3	0.5
Education (years)	14.1 ± 2.9	16.0 ± 3.3	**0.02**
Onset (bulbar / spinal)	4 / 16	—	
ALSFRS-R	40.7 ± 4.4	**—**	
FVC (% predicted)	92.5 ± 16.5	**—**	
Symptom Duration (months)	20.9 ± 21.2	**—**	
Disease progression rate	0.6 ± 0.7	**—**	
Finger tapping—right (/10 seconds)	45.2 ± 18.0	58.1 ± 8.2	**0.001**
Finger tapping—left (/10 seconds)	44.1 ± 15.1	53.4 ± 8.9	**0.006**
UMN score^†^	9.5 (1–21)	**—**	

Values are represented as Mean ± SD

^†^UMN score represented as Median (Range)

^‡^Differences in gender distribution computed using Chi-square;

p-values reaching significance at p<0.05 are represented in bold font.

### Clinical Assessment

Disease severity was inferred from the ALSFRS-R [26], a questionnaire that quantifies disability on 12 items assessing speech, swallowing, movement and respiratory function. Scores on the ALSFRS-R range from 0 to 48 with higher scores indicating lower disability. To account for variability in the progression of ALS, a disease progression rate was calculated as (48 –ALSFRS-R)/symptom duration [27]. Finger tapping scores, reflective of UMN functioning, for each limb was obtained for both patients and controls. Participants were instructed to tap with their index finger as fast as possible within 10 seconds. The number of taps was recorded and averaged over two administrations on each side (left and right, respectively). Neurological assessment for muscle stretch reflexes was performed on the patients by a neurologist (SK). A UMN disease burden score was calculated using an in-house adapted version of the Penn UMN score [24] as outlined in Table 2. The UMN score ranged from 0 to 32 with higher scores representing higher UMN disease burden. The score included presence or absence of hyperactive reflexes or clonus in each of the four limbs (0–7) and bulbar signs (0–2). Spasticity assessed using the modified Ashworth scale was incorporated into the UMN score for the respective limb with modified Ashworth rating of 3–4 adding 2 points, 1–3 adding 1 point and 0 adding no point [24]. A median UMN score was derived and patients scoring above the median were considered as UMN+ while those below the median were considered as UMN- groups for this study.

**Table 2:** UMN Scale adapted from Woo et al. [24].

UMN Domain	Score Range
*Bulbar*	
MSR jaw jerk reflex	0–1
Psuedobulbar affect	0–1
*Upper Extremity*	
Ashworth scale	0–2
MSR biceps	0–1
MSR triceps	0–1
Finger jerks	0–1
Hoffman's sign	0–1
Clonus	0–1
*Lower Extremity*	
Ashworth scale	0–2
MSR Quadriceps	0–1
MSR triceps surae	0–1
Adductors	0–1
Babinski's sign	0–1
Clonus	0–1
UMN score for each of left and right side	0–14
**Total UMN score**	0–30

UMN, Upper motor neuron; MSR, Muscle stretch reflex.

### MRI Protocol

Resting states fMRI (rs-fMRI) was acquired on a Varian Inova 4.7T scanner at the Peter S. Allen MR Research Centre. A single shot T2* weighted echo planar imaging sequence with TR/TE = 2500/19 ms, flip angle = 75°, matrix size = 67 × 80 points, field of view (FOV) = 240 mm^2^ was obtained. Thirty-six axial slices aligned to the AC-PC axis with slice thickness = 3 mm and voxel size = 3 × 3 × 3 mm were obtained. A total of 192 time points were acquired for each subject. Subjects were instructed to remain awake with their eyes closed without thinking about anything in particular during the scan. Anatomical scans were acquired with a high-resolution 3D magnetization prepared rapid gradient echo (MPRAGE) sequence with TR/TE/TI = 8.5/4.5ms/300ms, voxel size = 1 × 1 × 1 mm. matrix size = 256 x 200 points. An MPRAGE sequence with TR/TE/TI = 10.35/4 ms/300ms, voxel size = 1 x 1 x 1mm and matrix size = 256 × 200 points was acquired instead of the former sequence on a proportion of the participants. Both were interpolated to 0.5 × 0.5 × 1mm, matrix size 512 × 400 points.

### Data Analysis: Demographics and Clinical Assessment

Independent samples t-test was used to compute statistically significant differences in age, education and finger tapping scores between patients and controls. Pearson chi-square was computed to check for differences in distribution of gender between the two groups.

### MRI Analysis

The images were preprocessed and analyzed using Statistical Parametric Mapping version 8.0 (SPM8; www.fil.ion.ucl.ac.uk/spm) on MATLAB R2014a (Mathworks, Natick, MA, USA).

#### Preprocessing

The first 4 time-points of each subject’s scan were discarded to remove spin saturation effects. The remaining 188 time points of each subject were realigned to the first scan, normalized to the SPM8 EPI template and smoothed with a FWHM of 8 × 8 × 8 mm Gaussian kernel. The REST fMRI toolbox was used to apply band-pass filter at 0.01–0.1 Hz to exclude physiological noise at high and low frequencies.

#### Build ROI and extract time-course

Seeds for the DMN and the SMN were built using MarsBar on a standard MNI template [28] (http://marsbar.sourceforge.net/). The posterior cingulate cortex (PCC) was selected as a seed for the DMN [29] and a box ROI was built. Multiple regions in the precentral gyrus, postcentral gyrus and supplementary motor areas were selected for the SMN. Peak intensities published in previous research [30] were used as a mid-point for spherical ROIs (diameter of 10 mm). These multiple regions were combined to form a single seed for the SMN. The PCC and SMN seeds (S1 Fig) were used to extract time-courses for each subject and were used in the next step (whole brain voxel-wise correlations). Additionally, seeds were also built in multiple white matter (WM) regions and ventricles. Time courses from these seeds were extracted and used as regressors to reduce physiological noise due to cardiac rhythm and cerebrospinal fluid at the subject level.

#### Voxel-wise correlations

**Fig 1:**
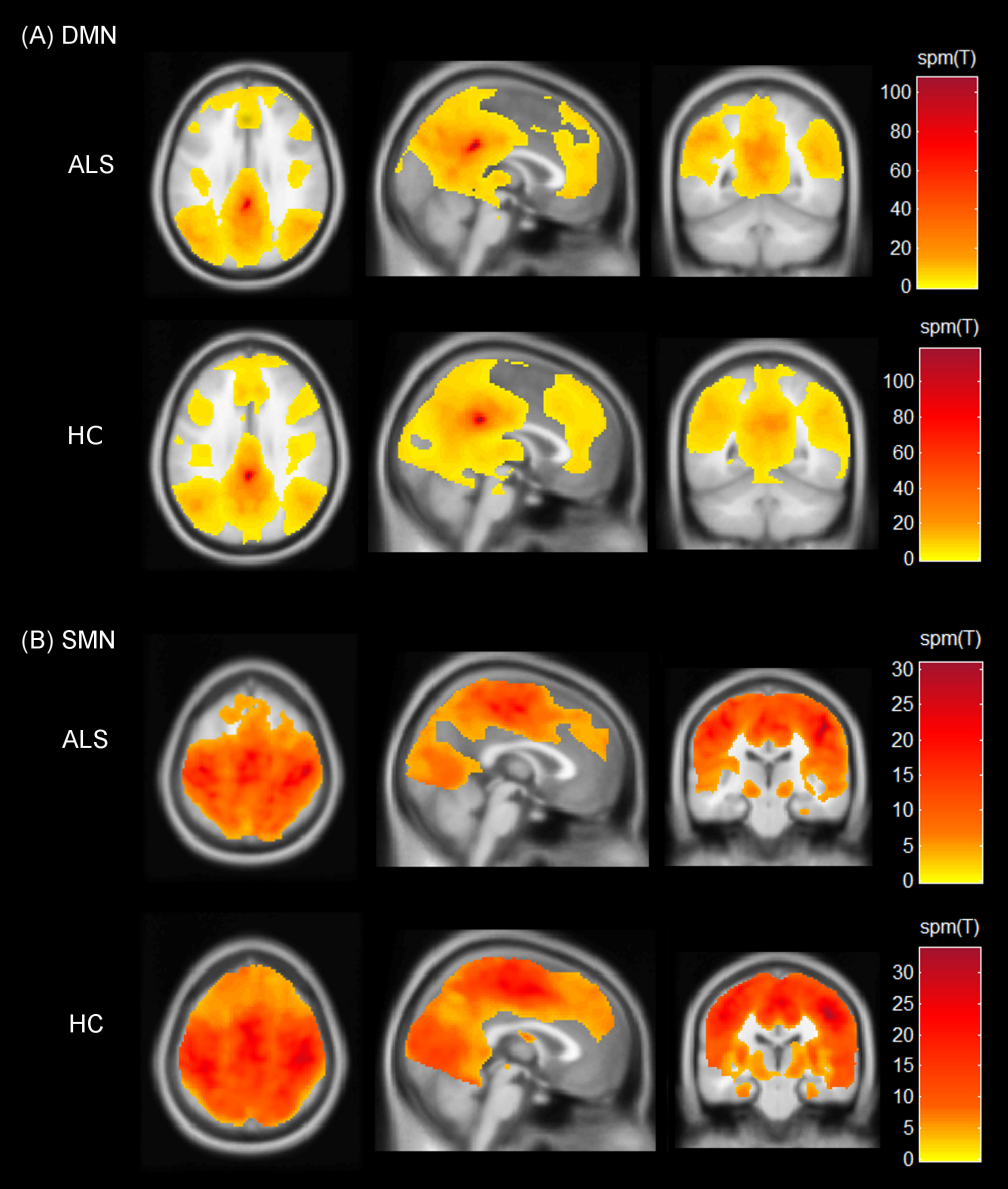
Spatial maps indicating the functional connectivity of the resting state networks. (A) Default mode network (DMN) and (B) Sensorimotor network (SMN). The spatial maps represent one-sample t-tests across patients and controls with threshold set at p<0.001 (cluster-size corrected at 90 voxels). Colour bars represent the spm(T) statistic for each map across the whole brain ranging from lowest (yellow) to the highest (dark red). Results are shown on Montreal Neurological Institute average brain in neurological convention.

Next, whole brain voxel-wise correlations for the DMN and the SMN using defined seed regions was performed for each subject. The time course of respective seed regions was correlated with each voxel individually for all subjects. The WM and ventricular time course, and motion parameters obtained during preprocessing, were used as regressors to reduce noise. The p-value threshold for the contrast was set at p<0.001 for the whole brain voxel-wise correlations for individual subjects for each of the RSNs. The spatial maps of the DMN and the SMN obtained for each group are shown in Fig 1. The contrast maps generated in this step reflect the connectivity of brain regions with the respective seeds and were used for further group analysis. The contrast maps obtained for each subject for the DMN and the SMN were visually inspected for artifacts. One patient and six controls were excluded due to poor visual quality. Thus, further group analysis and clinical correlations included 20 patients and 34 controls.

#### Group analysis

An independent samples t-test model was used to address hypothesis 1 (ALS vs controls) and hypothesis 2 (UMN+ vs UMN-). Dichotomization of patients into UMN+ and UMN- groups was determined by their median scores on the adapted UMN Scale (Table 2).

#### Clinical correlations

Multiple regression models were used to address hypothesis 3 (relating connectivity to the clinical variables).

Age was used as a covariate in all models for group analysis and clinical correlations to control for the effects of age-related brain changes. A primary threshold of p<0.001 was set for all models. Monte Carlo simulations were used to compute cluster size threshold (90 voxels) to correct for multiple comparisons at p<0.05.

## Results

### Demographics and Clinical Assessment

There were no significant differences in age between patients and controls (t = 0.5, *df* = 57, p = 0.6). Patients had significantly lower years of education as compared to controls (t = -2.4, *df =* 55, p = 0.02) and the median UMN Scale score of patients was 9.5. Pearson chi-square indicated a trend towards significance (χ^2^ = 3.2, *df* = 1, p = 0.08) in the distribution of gender between the two groups; controls had more females as compared to patients. Patients scored significantly lower than controls in the finger tapping test for both the left (t = -3.6, *df* = 53, p = 0.001) and the right (t = -2.9, *df* = 53, p = 0.007) sides.

### Group Analysis

*ALS vs*. *controls*: Independent samples t-test revealed no significant differences in connectivity the DMN and the SMN. *UMN+ vs*. *UMN-*: UMN scores were available for 16 patients and dichotomization classified 8 patients per group. No significant differences in connectivity were found between UMN+ and UMN-groups.

### Clinical Correlations

**Fig 2:**
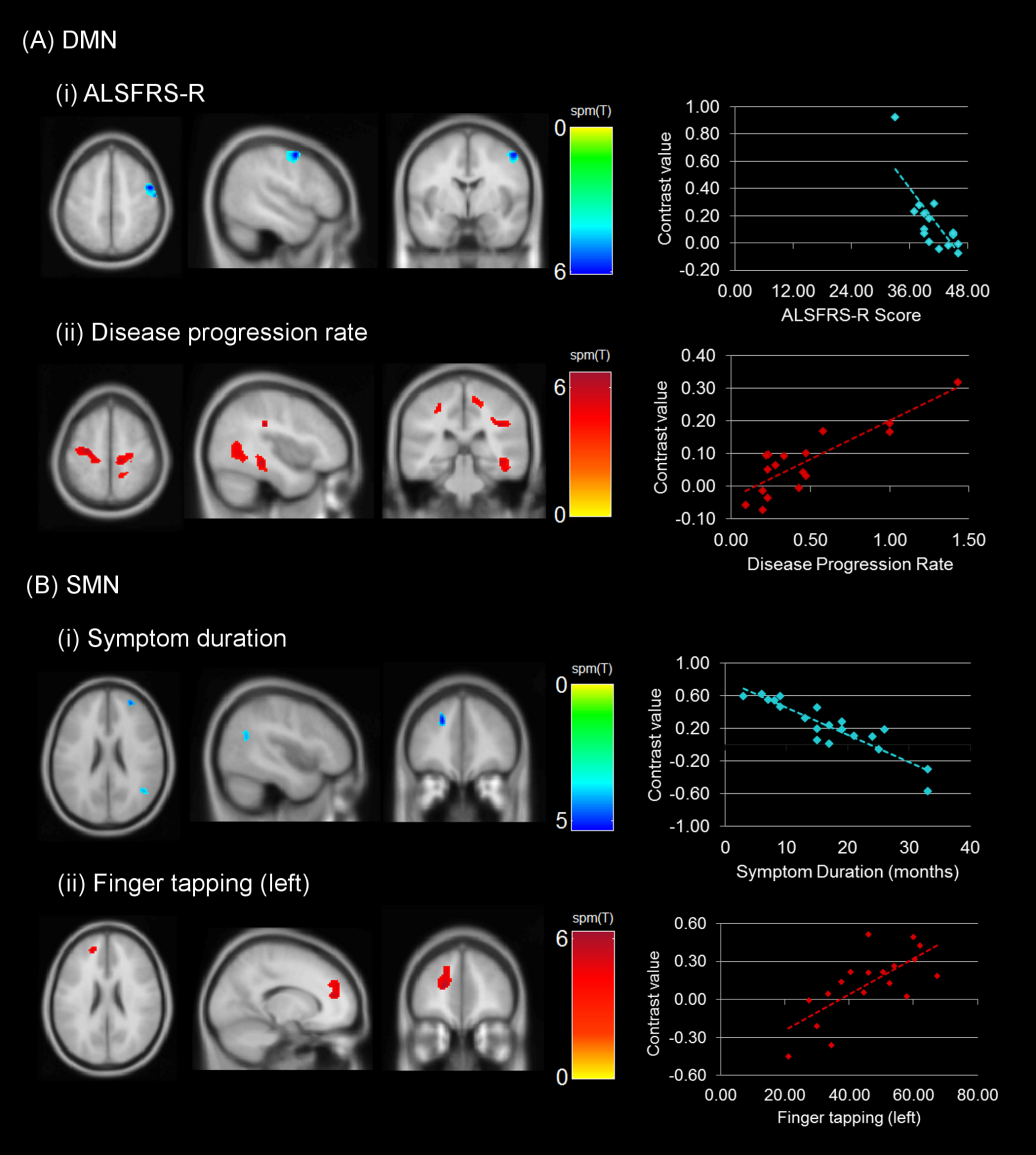
Regional correlations in connectivity with clinical measures. Images represent the correlations observed with the (A) DMN (ALSFRS-R and disease progression rate) and (B) SMN (symptom duration and finger tapping rate). A threshold of p<0.001 (cluster-size corrected at 90 voxels). The scatterplots represent the direction of associations between clinical measure (x-axis) and connectivity (contrast value on the y-axis). See Table 3 for details on the cluster extent and location.

Multiple regression models indicated significant associations between connectivity and clinical scores (Fig 2). Lower DMN connectivity was associated with higher ALSFRS-R scores in the right precentral gyrus (T = 6.02; p<0.001, cluster size corrected). On the contrary, higher DMN connectivity was associated with higher disease progression rates in widespread regions of the posterior aspect of the brain. Patients with higher disease progression showed increased connectivity with the PCC in bilateral precentral gyri, bilateral postcentral gyri, bilateral middle cingulate cortices (MCC) right PCC, and possible WM regions medial to the right posterior superior temporal gyrus. The peak intensities, corresponding MNI coordinates and the cluster extent are reported in Table 3.

**Table 3:** Significant regional associations between clinical variables and functional connectivity of the DMN and the SMN in patients. DMN connectivity was related to disease progression rate and inversely with disability as assessed by the ALSFRS-R. SMN connectivity was related to a motor task (finger tapping) and inversely with symptom duration.

Network	Clinical variable	Connectivity	MNI coordinates^†^	T	Cluster extent
DMN	ALSFRS-R	Lower connectivity in R precentral gyrus (BA 6) associated with higher ALSFRS-R scores	50 0 54	6.02	213
			56–10 50	5.39	
	Disease progression rate	Widespread clusters of higher connectivity associated with higher progression rate in:			
		L precentral gyrus	-28–16 58	6.37	659
		L MCC	-10–42 52	6.36	
		L postcentral gyrus	-26–40 46	6.31	
		L paracentral lobule	-18–26 64	5.29	
		WM region in R postcentral gyrus	42–30 32	6.60	447
		R precuneus	12–48 62	6.35	
		R MCC	10–40 48	4.67	
		R precentral gyrus	24–18 74	5.06	445
		R posterior-medial frontal	12–18 54	4.34	
		R PCC	8–44 22	5.53	100
		Possible WM region in R posterior temporal regions	42–34–10	5.97	737
SMN	Symptom Duration	Lower connectivity associated with higher symptom duration in the:			
		anterior region of R middle frontal gyrus	30 54 20	5.30	113
		anterior region of L superior frontal gyrus (including BA 9)	-20 38 34	5.30	104
		posterior region of R middle temporal gyrus	48–60 18	4.59	102
	Finger tapping (left)	Higher connectivity associated with higher tapping scores in the anterior region of the L superior frontal gyrus	-20 48 14	6.27	256

MCC, middle cingulate cortex;

PCC, posterior cingulate cortex;

^†^MNI coordinates derived using SPM Anatomy toolbox [31] representing the location of the peak voxel intensity (T) for each significant cluster.

More than one T-value for a cluster represents peak intensities at least 8mm apart.

Lower SMN connectivity was found to be associated with higher symptom duration in three clusters with peak intensities in the right anterior region of the middle frontal gyrus (T = 5.30), anterior region of the left superior frontal gyrus (T = 5.30) and posterior region of the right middle temporal gyrus (T = 4.59). Higher SMN connectivity was associated with higher left finger tapping scores in the anterior regions of the left superior frontal gyrus (T = 6.27; Fig 2B, Table 3).

## Discussion

Our study aimed at evaluating DMN and SMN connectivity changes in patients with ALS and to identify connectivity changes that may contribute in predicting clinical features. Our results indicate no significant changes in connectivity between [32] ALS patients and healthy controls, and (2) UMN+ and UMN- patients. (3) We report significant associations between RSN connectivity and clinical variables that may provide insight into factors that predict functional changes.

One previous study [17] is in line with our findings and reports no significant differences in DMN connectivity in patients with ALS. However, the authors of the same paper report decreased connectivity in the premotor cortex for the SMN [17]. In contrast to our study some studies report decreased DMN connectivity in the PCC [16], orbitofrontal cortex [12] and decreased SMN connectivity in the premotor areas [12, 15, 17, 18]. A few studies also report increased connectivity in the premotor cortices in patients [19, 20]. Several factors may contribute to the inconsistencies observed in RSN connectivity in ALS. It is well established that the disease course is variable amongst patients, and patient characteristics could have differed significantly between studies, including age of onset, disease severity and rate of progression. Technical aspects such as acquisition parameters and data processing methods may contribute to differences in findings [13].

We found increased DMN connectivity in the right precentral gyrus associated with greater disability as indicated by reduced ALSFRS-R scores. This is not in accordance with previous literature in the field; studies either report decreased functional connectivity to be associated with lower ALSFRS-R scores [12, 18, 19] or report no significant associations between functional connectivity and ALSFRS-R [13, 14, 16, 21, 23, 33]. We also report that higher DMN connectivity between bilateral motor cortices, precuneus, postcentral gyrus, right posterior temporal regions and the PCC was associated with higher disease progression rate in patients indicating more widespread changes in functional connectivity in faster progressing patients. Our finding is supported by one previous study indicating higher functional connectivity in fast progressors predominantly in the motor and sensorimotor cortices [23]. Positron emission tomography (PET) studies suggest a loss of inhibitory interneurons in the corticospinal tract [34] associated with higher connectivity and more advanced disease. It has been proposed that this loss of inhibitory interneurons in the motor cortices may also result in higher baseline activity [20]. Our DMN findings collectively suggest that ALS patients with greater disability and faster progression show higher functional connectivity at rest that could be speculated to be due to the loss of inhibitory interneurons.

SMN connectivity was found to be decreased between the bilateral frontal regions of the brain, posterior region of the right middle temporal gyrus and the SMN seed in association with higher symptom duration. A previous study reports that lower regional homogeneity (ReHo) was associated with longer disease duration [18]. ReHo is another voxel-based measure of brain activity to evaluate the synchronization between the time series of a voxel and its neighbours [35]. This indicates that patients who have had ALS symptoms for a longer duration may eventually show decline in connectivity which may not be evident during the initial phase of the disease. This is supported by a study reporting lower SMN connectivity in moderate to advanced stages on ALS [19]. On the other hand, hyperexcitability of motor cortices have been reported in the early stages of ALS [36, 37] and may appear a few months prior to the onset of symptoms [38]. While studies speculate the exact onset of ALS, evidence from supporting presymptomatic and interneuronopathy related changes suggest that our finding of higher functional connectivity may be predominant in patients short symptom duration [12] and these spontaneous activations at rest may decrease with longer symptom duration and further reflecting extent of interneuropathy.

We also report that connectivity between the left superior frontal gyrus and the SMN seed increases with higher left finger tapping. Previous studies using task-based fMRI to study finger tapping, report an increase in ipsilateral activation of premotor areas in ALS patients as compared to controls [39]. Additionally, studies have also reported the recruitment of cerebellar regions in ALS as compared to controls while performing task-based motor activity [40]. Our findings suggest that additional ipsilateral frontal areas may be associated with the SMN as a result of loss of cortical interneurons to inhibit the spontaneous activations at rest.

To our best knowledge, this is the first report exploring UMN burden and functional connectivity in ALS. The novel finding is that functional connectivity is not dependent on UMN burden as analyzed by sub-grouping patients into UMN+ and UMN- groups. The UMN pathway is classically described as motor neurons originating in the motor regions of the cerebral cortex that project to the brain stem and the lower motor neurons. The RSN activity may not be affected with the degeneration of these UMNs which do not have intracerebral connections as would association and commissural fibres. On the other hand, other MRI measures such DTI may be more sensitive to this loss as they primarily reflect the structural integrity of white matter tracts irrespective of their connections.

The presence of associations between clinical parameters and RSN connectivity supports aspects of pathology reported in previous literature as well as highlights the possibility of individual differences in disease course. While it is promising to see the association of RSN connectivity with clinical parameters, whether this association implies a functional reorganization that predicts disease-specific parameters would require further investigation. It would be interesting to longitudinally explore whether such changes are indeed re-organization and if the changes can further predict prognosis or survival in ALS patients.

Our study employed seed based correlation as opposed to the ICA approach reported by previous studies in ALS. This provided an advantage of studying voxel-wise correlations of the brain and the seed region for each network of interest [19]. We recruited patients within 2 years of symptom durations and with relatively lower disease severity, thereby allowing us to explore functional changes associated with factors such as shorter symptom duration. Further, we employed a stringent threshold to analyse our results and correct for multiple comparisons. This was aimed at addressing concerns raised by a recent study regarding cluster thresholding primary thresholds at p<0.05 or p<0.01 [41]. It was indicated that statistical methods to correct for multiple comparisons may not satisfy the required criteria as expected at such lenient primary thresholds and may show at least 9–12% probability of type I and type II errors, while more stringent primary thresholds (p<0.001) coupled with a secondary threshold to control for multiple comparisons would reduce the risk of type I and type II errors to 5 percent.

Nevertheless, our study is not without limitations. We do not report structural changes that may be associated with functional connectivity. Additional multi-modal analysis would provide further insight into the results obtained and may prove to be more comprehensive in understanding functional changes in ALS. Participants in our study were trending towards significance in the distribution of gender between the two groups. While gender differences in RSN connectivity has been reported to be weak by previous studies [30] including gender matched participants reduces the chance for variability. Moreover, the cognitive profile of the patients is not included in the current study. Neuropsychometric testing was performed on only 10 patients. The small sample size provided insufficient power for statistical analysis.

## Conclusions

We report significant associations between RSN connectivity and clinical variables for the DMN and the SMN. DMN connectivity was increased in those with greater disability and those with a faster progression rate, and SMN connectivity was reduced with greater clinical motor impairment. It appears that although RSN alterations in ALS may not reach statistical significance in comparison with controls, the association of increased or decreased connectivity with clinical parameters suggests underlying functional changes in the cortex that may reflect loss of inhibitory neurons. It would be of interest to employ multi-modal imaging analysis on a larger sample of participants and include additional RSNs such as the executive control network in the future for a comprehensive understanding of functional changes in ALS.

## Supporting Information

S1 Fig Seed regions used to extract time-course for resting state analysis. Images display the seeds used for the (A) Default mode network (DMN) and (B) Sensorimotor network (SMN). Seeds are represented on the Montreal Neurological Institute averaged brain.
(PDF)
