# Supplementary material for: Investigating Default Mode and Sensorimotor Network Connectivity in Amyotrophic Lateral Sclerosis
Source: PLoS One. 2016 Jun 20;11(6):e0157443. doi: 10.1371/journal.pone.0157443 (PMC4913931; doi:10.1371/journal.pone.0157443)
Supplement: S1 Fig — Images display the seeds used for the (A) Default mode network (DMN) and (B) Sensorimotor network (SMN). Seeds are represented on the Montreal Neurological Institute averaged brain. (PDF) [file pone.0157443.s001.pdf]

S1 Fig. Seed regions used to extract time-course for resting state analysis. Images display the seeds used for the (A) Default mode network (DMN) and (B) Sensorimotor network (SMN). Seeds are represented on the Montreal Neurological Institute averaged brain.
